# Measles Outbreak Associated with Adopted Children from China — Missouri, Minnesota, and Washington, July 2013

**Published:** 2014-04-11

**Authors:** Edith N. Nyangoma, Christine K. Olson, Stephen R. Benoit, John Bos, Chas DeBolt, Meagan Kay, Krista Rietberg, Azadeh Tasslimi, Douglas Baker, Xinwen Feng, Susan Lippold, Sena Blumensaadt, Christopher Schembri, Arnold Vang, Heather Burke, Gregory Wallace, Weigong Zhou

**Affiliations:** 1 EIS officer, CDC; 2 Division of Global Migration and Quarantine, National Center for Emerging and Zoonotic Infectious Diseases, CDC; 3 Missouri Department of Health and Senior Services; 4 Washington State Department of Health; 5 Public Health–Seattle and King County, Washington; 6 Guangdong International Travel Healthcare Center, China; 7 Division of Viral Respiratory Diseases, National Center for Immunization and Respiratory Diseases, CDC

On July 5, 2013, CDC was notified of two cases of laboratory-confirmed measles in recently adopted children from an orphanage in Henan Province, China. To find potentially exposed persons, CDC collaborated with state and local health departments, the children’s adoption agency, and airlines that carried the adoptees. Two additional measles cases were identified, one in a family member of an adoptee and one in a third adopted child from China. To prevent further importation of measles, CDC worked with health officials in China, including “panel physicians” contracted by the U.S. Department of State to conduct the overseas medical examinations required for all immigrants and refugees bound for the United States. The following measures were recommended: 1) all adoptees examined at panel physician facilities should be screened for fever and rash illness, 2) measles immunity should be ensured among all adoptees from Henan Province who are scheduled for imminent departure to the United States, and 3) all children at the orphanage in Henan Province should be evaluated for measles. This report summarizes the results of the outbreak investigation and underscores the importance of timely routine vaccination for all international adoptees.

## Investigation and Public Health Response

A boy and a girl, both aged 2 years and with cerebral palsy, were in the process of being adopted by families in the United States, but became ill in China before traveling to the United States. The boy (child A) developed rhinorrhea and cough on June 24. At the time of his immigration medical examination by a panel physician on June 29, the boy was found to have a rash on his neck. Because he was afebrile and had no other symptoms or signs, the rash was diagnosed as contact dermatitis. By the next day, the rash began on his head and spread to his trunk and extremities, and he developed a fever. The girl (child B) was noted to be febrile on June 29 during her immigration medical examination, but no other symptoms or signs were present. Two days later, the panel physician was told by her adoptive parents that the girl was afebrile and doing well. However, investigators later learned that on June 29 the girl had developed cough, fever, and conjunctivitis, and on July 1 she had developed a rash on her face and neck.

On July 4, both ill children traveled on different flights to the United States. They were hospitalized shortly after arrival in Washington and Missouri, respectively. Measles was confirmed in both children by positive immunoglobulin M (IgM) serology and polymerase chain reaction (PCR), and both were placed on isolation precautions. Neither child had documented measles vaccination, and their adoptive parents had executed affidavits, consistent with current policy, for exemption from the vaccine requirements for immigration until after their arrival in the United States.

State public health authorities notified CDC’s Division of Global Migration and Quarantine (DGMQ) because both children were contagious* during travel. DGMQ, working with U.S. Customs and Border Protection, obtained passenger and crew manifests and contact information from the airlines to investigate potential exposure of persons on flights with the ill children. A total of 83 crew members and passengers (those seated in the same row, two rows in front of, and two rows behind the ill children; infants in arms seated anywhere on either plane; and crew members who served passengers in the same cabin as either ill child)† were identified as potentially exposed to measles. Contact information was available for 74 passengers and crew members from 20 states. Of the 29 passengers for whom follow-up data were available, two were found to be susceptible to measles, and one received postexposure immunoglobulin. No secondary measles cases associated with these flights were reported to CDC.

Local health officials conducted contact investigations with family members of the two ill adopted children, health-care personnel and other persons in health facilities where the children were treated, and two other U.S. families of recently adopted children who were in contact with the ill children in China. Contacts were interviewed about their travel history, presence of fever and rash, and measles immunity, either through documented vaccination or serologic tests. Contacts with no evidence of measles immunity were recommended to receive either postexposure vaccination with measles, mumps, and rubella (MMR) vaccine within 3 days of initial measles exposure or immunoglobulin administration within 6 days of initial measles exposure. Of 132 persons contacted, six were found to be susceptible to measles; three received postexposure immunoglobulin.

Two additional cases of measles were identified. Child C, aged 2 years in Minnesota, was adopted from China at the same time as child A and child B. Although child C was adopted from a different orphanage and traveled on different flights, he was exposed to the two infected children during the emigration process in China. Child C was asymptomatic and not contagious during travel. He arrived in the United States on July 4, developed a fever on July 10, rash on July 14, and tested IgM-positive for measles on July 16. This child also had cerebral palsy and no documentation of measles vaccination. The child’s family members were fully immunized, and no secondary cases were reported.

A fourth case was identified in the adoptive mother, aged 39 years, of child B. Although she reported a history of vaccination against measles, she developed signs and symptoms compatible with modified measles. On July 14 (2 weeks after symptom onset in child B), she developed a rash on her face and neck that lasted 4 days, but she had no fever, respiratory, or ocular symptoms. PCR and IgM tests performed on July 19 were positive for measles. An investigation of 19 contacts in this family revealed no additional cases.

### Discussion

Although endemic measles was declared eliminated from the United States in 2000, sporadic outbreaks still occur as a result of importations from areas where measles is endemic (1). Recent reported outbreaks have been associated with international adoptions, including two outbreaks among adoptees from China in 2004 and 2006 (2,3). This report documents a cluster of imported measles cases among adoptees from China in July 2013.

China is the leading country of origin for internationally adopted children in the United States, contributing 30% (2,696) of foreign-born adoptions in 2012 (4). Through improved surveillance and enhanced nationwide vaccination campaigns using a 2-dose routine measles immunization schedule administered at 8 months and 18 months, with >90% measles vaccination coverage, cases of measles have significantly declined in China, from 2.8 per 100,000 in 2010 to 0.5 per 100,000 in 2012 (5). However, in the first months of 2013, the number of measles cases in China rose to an incidence of 2.7 per 100,000 (6).

Adoption of children from countries with high rates of vaccine-preventable diseases presents a challenge to the control of communicable diseases in the United States. Since 1996, the U.S. Department of State has required all persons seeking a U.S. immigrant visa to show proof of vaccination for certain vaccine-preventable diseases, including measles.§ However, internationally adopted children aged ≤10 years are exempted from this requirement if the adopting parent(s) sign an affidavit indicating that the child will receive vaccination within 30 days of entry into the United States.¶ A significant public health risk is posed before, during, and after travel to the United States when parents choose not to vaccinate their children before leaving the country of origin. Children who are malnourished or have concomitant conditions, which are common among foreign-born adoptees, are especially vulnerable to severe forms of vaccine-preventable diseases (1). Parents who choose to sign the affidavit should ensure that adoptees receive age-appropriate vaccines, as recommended by the Advisory Committee on Immunization Practices,** as soon as possible following entry into the United States.

In this outbreak, all measles cases among adoptees were in unvaccinated children aged 2 years with cerebral palsy. Previous studies have shown that children with cerebral palsy are at risk for incomplete or delayed immunization, possibly as a result of the misconception that the MMR vaccine is associated with harmful neurologic side effects (7,8). Cerebral palsy is not a contraindication for MMR vaccination. Health-care providers should ensure that all children, including those with cerebral palsy, receive timely, age-appropriate routine immunizations, as recommended by the World Health Organization†† and the country of origin, unless contraindicated by medical conditions (e.g., history of anaphylactic reaction to a vaccine component).

Two of the ill children reported in this cluster were symptomatic and contagious en route to the United States, potentially exposing their families, many travelers, and hospital patients and staff members to measles. Had they been identified as infectious with measles before travel and prevented from boarding, subsequent contact investigations by health officials and the substantial costs associated with control of the outbreak could have been avoided. More importantly, routine vaccinations according to recommended schedules likely would have averted this outbreak. Public health and adoption agencies should continue to promote appropriate vaccinations for adoptees and their adoptive families

What is already known on this topic?

The United States eliminated domestic transmission of measles in 2000. Unvaccinated travelers to and from parts of the world where measles is still endemic continue to pose a risk for acquiring and transmitting the disease in the United States.

What is added by this report?

Two cases of measles were diagnosed in adopted children from China in July 2013, shortly after the children arrived in the United States. CDC collaborated with state and local health departments, the children’s adoption agency, and the airlines on which the adoptees traveled to find potentially exposed persons. Two additional measles cases were identified, one in a family member of an adoptee and one in another adopted child recently arrived from China. None of the three children had been vaccinated against measles.

What are the implications for public health practice?

Foreign-born adopted children can be the source of imported measles. Efforts to ensure that adopted children receive safe and timely vaccination, as recommended by the World Health Organization and the country of origin, are critical to prevent similar outbreaks.

## Footnotes

* The contagious period for measles is considered to be from 4 days before to 4 days after the onset of rash.

† Additional information available at http://www.cdc.gov/quarantine/contact-investigation.html and http://www.cdc.gov/quarantine/air/managing-sick-travelers/commercial-aircraft/measles.html.

§ Illegal Immigration Reform and Immigrant Responsibility Act of 1996. Pub. L. No. 10-208, 110 Stat. 3009 (September 30, 1996).

¶ The International Adoption Simplification Act of 2010. Pub. L. No. 111-287, 124 Stat. 3058 (November 30, 2010).

** Available at http://www.cdc.gov/vaccines/acip.

†† Available at http://www.who.int/immunization/policy/en.

## References

[1] Atkinson W, Wolfe S, Hamborsky J, CDC (2012). Measles [Chapter 12]. Epidemiology and prevention of vaccine-preventable diseases.

[2] CDC (2004). Multistate investigation of measles among adoptees from China—April 9, 2004. MMWR.

[3] CDC (2007). Measles among adults associated with adoption of children in China—California, Missouri, and Washington, July–August 2006. MMWR.

[4] US Department of State (2013). FY 2012 annual report on intercountry adoption.

[5] World Health Organization (2014). Western Pacific Region. Measles in China fact sheet.

[6] Ma C, Hao L, Zhang Y (2014). Progress towards measles elimination in China. Bull World Health Organ.

[7] Greenwood VJ, Crawford NW, Walstab JE, Reddihough DS (2013). Immunization coverage in children with cerebral palsy compared with the general population. J Pediatric Child Health.

[8] Mäkelä A, Nuorti JP, Peltola H (2002). Neurologic disorders after measles-mumps-rubella vaccination. Pediatrics.

