# Erratum

**Published:** 2014-05-02

## Vol. 63, No. 14

In the report, “Measles Outbreak Associated with Adopted Children from China — Missouri, Minnesota, and Washington, July 2013,” an error occurred on page 302 in the fifth sentence of the second paragraph of the right column. The sentence should reads as follows: “He arrived in the United States on July 4, developed a fever on July 10, rash on July 14, and tested **PCR**-positive for measles on July 16.”

